# Comparative Genomic Analysis of Two Serotype 1/2b *Listeria monocytogenes* Isolates from Analogous Environmental Niches Demonstrates the Influence of Hypervariable Hotspots in Defining Pathogenesis

**DOI:** 10.3389/fnut.2016.00054

**Published:** 2016-12-21

**Authors:** Aidan Casey, Kieran Jordan, Aidan Coffey, Edward M. Fox, Olivia McAuliffe

**Affiliations:** ^1^Teagasc Food Research Centre, Fermoy, Ireland; ^2^Department of Biological Sciences, Cork Institute of Technology, Bishopstown, Ireland; ^3^CSIRO Agriculture and Food, Werribee, VIC, Australia

**Keywords:** comparative genomic analysis, *Listeria monocytogenes*, pathogenesis, hypervariable hotspots, attenuated virulence, stress survival islet, LIPI-3, DPC6895, serotype 1/2b

## Abstract

The vast majority of clinical human listeriosis cases are caused by serotype 1/2a, 1/2b, 1/2c, and 4b isolates of *Listeria monocytogenes*. The ability of *L. monocytogenes* to establish a systemic listeriosis infection within a host organism relies on a combination of genes that are involved in cell recognition, internalization, evasion of host defenses, and *in vitro* survival and growth. Recently, whole genome sequencing and comparative genomic analysis have proven to be powerful tools for the identification of these virulence-associated genes in *L. monocytogenes*. In this study, two serotype 1/2b strains of *L. monocytogenes* with analogous isolation sources, but differing infection abilities, were subjected to comparative genomic analysis. The results from this comparison highlight the importance of accessory genes (genes that are not part of the conserved core genome) in *L. monocytogenes* pathogenesis. In addition, a number of factors, which may account for the perceived inability of one of the strains to establish a systemic infection within its host, have been identified. These factors include the notable absence of the *Listeria* pathogenicity island 3 and the stress survival islet, of which the latter has been demonstrated to enhance the survival ability of *L. monocytogenes* during its passage through the host intestinal tract, leading to a higher infection rate. The findings from this research demonstrate the influence of hypervariable hotspots in defining the physiological characteristics of a *L. monocytogenes* strain and indicate that the emergence of a non-pathogenic isolate of *L. monocytogenes* may result from a cumulative loss of functionality rather than by a single isolated genetic event.

## Introduction

*Listeria monocytogenes* is a Gram-positive, facultatively anaerobic food-borne pathogen, and is the causative agent of the bacterial disease listeriosis in humans and animals. Recent figures demonstrate that approximately 99% of all human listeriosis cases arise due to the consumption of contaminated food produce (1), with serotypes 1/2a, 1/2b, 1/2c, and 4b implicated as the source of infection in 95% of these cases (2). Its psychrotrophic nature coupled with its tolerance of low pH and high salt concentrations (3) allows the bacterium to survive in refrigerated foods and reach levels required for human infection, if the food can support growth. *L. monocytogenes* is also commonly found in farm environments and silage in particular (4), and as such contaminated feeds represent a similar vector for food-borne transmission of the bacterium to animals used in food production. Its ability to cause a systemic infection in humans and animals alike is reliant on a combination of physical attributes, including resistance to environmental stresses and a capacity for virulence and survival within the host.

Traditionally, genetic relationships between *L. monocytogenes* strains are elucidated either by pulsed-field gel electrophoresis (PFGE) involving macrorestriction of genomic DNA to generate an associated DNA fingerprint (5) or by multilocus sequence typing (MLST) where specific sequences from a number of housekeeping genes are analyzed (6). These approaches are limited, however, in that they provide little insight into the pan genome of *L. monocytogenes* isolates. Comparative genome analysis has emerged as a robust tool for evaluating underlying genetic properties of bacterial strains, such as their evolutionary relationships, pathogenic potential, antibiotic resistances, and niche adaptation capabilities. With regard to *Listeria*, comparative genomics has proven to be particularly effective in determining the basis behind a number of observed phenotypic characteristics of *L. monocytogenes*, including the putative identification of many virulence genes responsible for *L. monocytogenes* pathogenesis on the basis of their relative absence in strains of the non-pathogenic *Listeria innocua* (7–9). Also, recent comparative analysis of two persistent *L. monocytogenes* strains that were isolated from separate fish processing plants almost 6 years apart (10) identified an extremely close relationship between their genomes. As such, it was proposed that strains with specific genetic traits may be selected for within a given environmental niche, providing a potential insight into the mechanisms of persistence of *L. monocytogenes*. Persistence is defined as the regular re-isolation of a given strain from the same environment over the course of several months or years. Comparative genomics has also been used to analyze *L. monocytogenes* isolates associated with listeriosis outbreaks (11, 12), to understand the unique genomic properties harbored by these strains contributing to systemic infection, and to determine the most efficient manner in which to analyze the epidemiological traits of future outbreak strains (13).

From an evolutionary perspective, one particular study involving a range of *L. monocytogenes* genomes of differing lineage and serotype demonstrated that this bacterial species, like others, has a highly conserved set of genes shared by all sequenced strains known as the “core genome” (14). While this core genome is common to all strains, subtyping methods (such as PFGE, MLST, and ribotyping) have demonstrated that examined *L. monocytogenes* isolates form a structured population consisting of a number of different evolutionary lineages (15). The majority of tested strains of serotypes 1/2a, 1/2b, 1/2c, 3a, 3b, 3c, 4b, and 4e cluster to evolutionary lineages I and II. Flagellar type “a” isolates such as serotypes 1/2a and 3a cluster to lineage II along with serotype 1/2c and 3c isolates, while flagellar type “b” isolates such as the 1/2b, 3b, and 4b serotypes all cluster within lineage I along with serotype 4d and 4e isolates (15). Two other evolutionary lineages have also been discovered and characterized. Lineage III contains serotype 4a, 4c, and a small number of 4b isolates (16) and represents a sister group to lineage I (15). Lineage IV, which was originally thought to represent a subgroup of lineage III (IIIB), is the most recently discovered (17), though only a limited number of isolates belonging to this lineage have been characterized to date. In general, the genomes of lineage I strains of *L. monocytogenes* (serotypes 1/2b, 3b, 4b, and 7) share a much higher degree of sequence similarity and exhibit a much lower degree of recombination than their lineage II and III counterparts (18, 19). Indeed, lineage I strains of *L. monocytogenes* predominantly differ from one another only in terms of their serotype, sequence type, prophage compositions, and a small fraction of chromosomal genes (12–23% of the total genome) that are collectively known as the accessory genome (14). Accessory genes are not as highly conserved as the core genes and in many cases can be strain-specific. Furthermore, while these accessory genes are located throughout the *L. monocytogenes* genome, their distribution is not entirely random. In certain chromosomal regions, accessory gene accumulations occur as a result of prophage acquisition (14), while other regions exhibit a non-random accumulation of these genes and are therefore denoted “hypervariable hotspots,” with nine such genomic regions recently defined in *L. monocytogenes* (19).

In this study, the genomes of two serotype 1/2b isolates of *L. monocytogenes* were subjected to comparative analysis in order to determine if there is a link between their core and accessory genome contents and their phenotypic characteristics. The two strains differed in their infection abilities. One of the isolates, strain DPC6895, was incapable of establishing a systemic infection within its animal host, despite it representing one of the four *L. monocytogenes* serotypes responsible for the vast majority of listeriosis cases (2, 20). Instead, this isolate caused a subclinical infection (21), and such subclinical infections generally go undetected, resulting in a potential public health hazard. On the other hand, strain FSL J2-064 did cause a systemic infection within its animal host. The aim of this research was to focus on a broad comparison of genes responsible for infection, intracellular survival, and proliferation within the host, in an attempt to discover a genomic basis for the perceived attenuation of pathogenesis in strain DPC6895 compared to strain FSL J2-064, and to evaluate the importance of the accessory genome in *L. monocytogenes* virulence and disease manifestation.

## Materials and Methods

### Input Strains for Comparative Analysis

The two *L. monocytogenes* strains examined in this study were of the 1/2b serotype. Strain DPC6895 was originally isolated from raw milk expressed by a cow with subclinical bovine mastitis (20, 21), while strain FSL J2-064 is a bovine clinical isolate (22, 23), but of a ribotype (or restriction digest fingerprint) that is also commonly found among food isolates (DUP-1052), and is associated with human disease (24). The genomes of both strains are available from public databases. The Whole Genome Shotgun project for *L. monocytogenes* strain DPC6895 was deposited at DDBJ/EMBL/GenBank and is available for download under the accession number LABG00000000. The version described in this paper is version LABG01000000. The genome sequence of *L. monocytogenes* strain FSL J2-064 is available from GenBank under the accession number NC_021824.

### Identification of Strain-Specific Genes in Each of the Input Genomes

Whole genome comparisons were undertaken using BLAST Ring Image Generator (25) and Mauve (26), in order to visually identify unique genomic regions belonging to each of the strains. Genes within these regions were then confirmed to be strain-specific to each particular isolate through BLAST comparisons of their translated protein sequences against the genome of the other isolate, using RAST (27, 28).

### Detection of Clustered Regularly Interspaced Short Palindromic Repeat (CRISPR)/CRISPR-Associated (Cas) Systems and Prophage Identification

Clustered regularly interspaced short palindromic repeat clusters in each genome were identified using CRISPRFinder (29), with flanking sequences of these clusters subsequently scanned for the presence Cas gene sequences. Viable and cryptic prophages within each of these genomes were detected using the PHAST tool (30).

### Linear Comparisons and Identification of Hypervariable Hotspots

Linear comparisons of genes and gene clusters were prepared with Artemis (31) and subsequently visualized using the EasyFig software (32). Hypervariable hotspot locations in each genome were determined *via* BLAST analysis using conserved core gene identifiers and previously defined hypervariable hotspot locations for *L. monocytogenes* strain SLCC2755, which was used as a reference (19).

## Results and Discussion

### General Features of the *L. monocytogenes* Serotype 1/2b Strains

The main features of both strains used in this study (Table 1), as well as the locations of their respective hypervariable hotspots (Table S1 in Supplementary Material) are shown. Both genomes were similar in length (2.9–3.0 Mb) and had a G + C content of 37.8–38%, which is within the range typically observed for strains of *L. monocytogenes*. Neither of these strains contained any plasmids.

**Table 1:** **General features of the *Listeria monocytogenes* input strains**.

	DPC6895	FSL J2-064
Origin of isolate	Bovine	Bovine
Genome length	2,919,539	2,943,218
Contigs	9	1
G + C content	37.80	38.00
No. of coding sequences (CDS)	2,874	2,828
No. of tRNA genes	54	58
No. of plasmids	0	0

**Nucleotide sequence blast (BLASTn) between input isolates (% query coverage, % identity, *E*-value)**		

	**DPC6895^a^ (subject)**	**FSL J2-064 (subject)**
DPC6895^a^ (query)		(96%, 99%, 0.0)
FSL J2-064 (query)	(95%, 99%, 0.0)	

*^a^Genome is not closed*.

### Comparative Genomic Analysis of Strains DPC6895 and FSL J2-064

**Figure 1:**
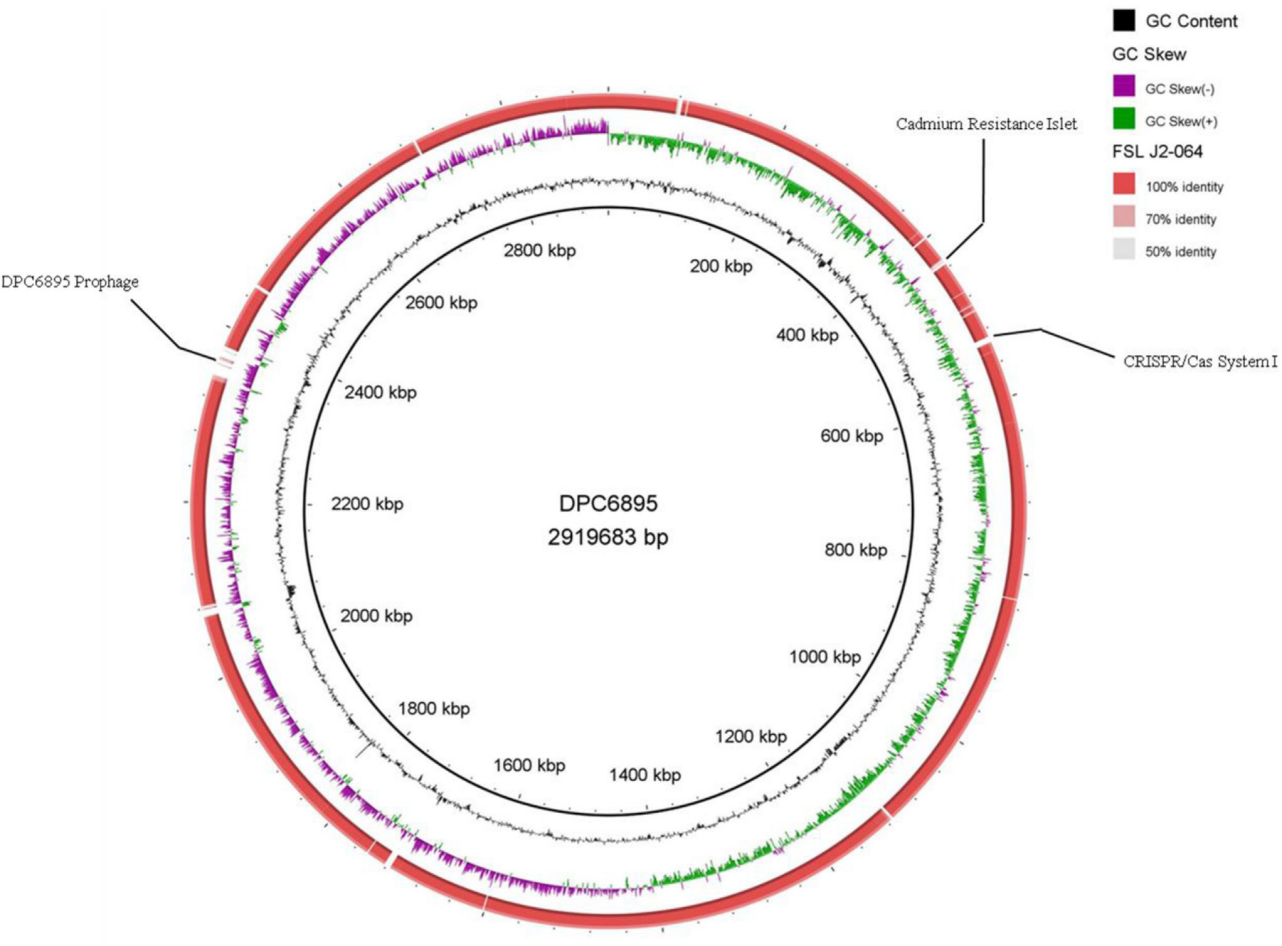
**Circular map of *Listeria monocytogenes* strain FSL J2-064, using strain DPC6895 as a reference genome**. The inner ring denotes the DPC6895 genome with corresponding genetic coordinates. The outer ring denotes the genome of strain FSL J2-064 (red).

**Figure 2:**
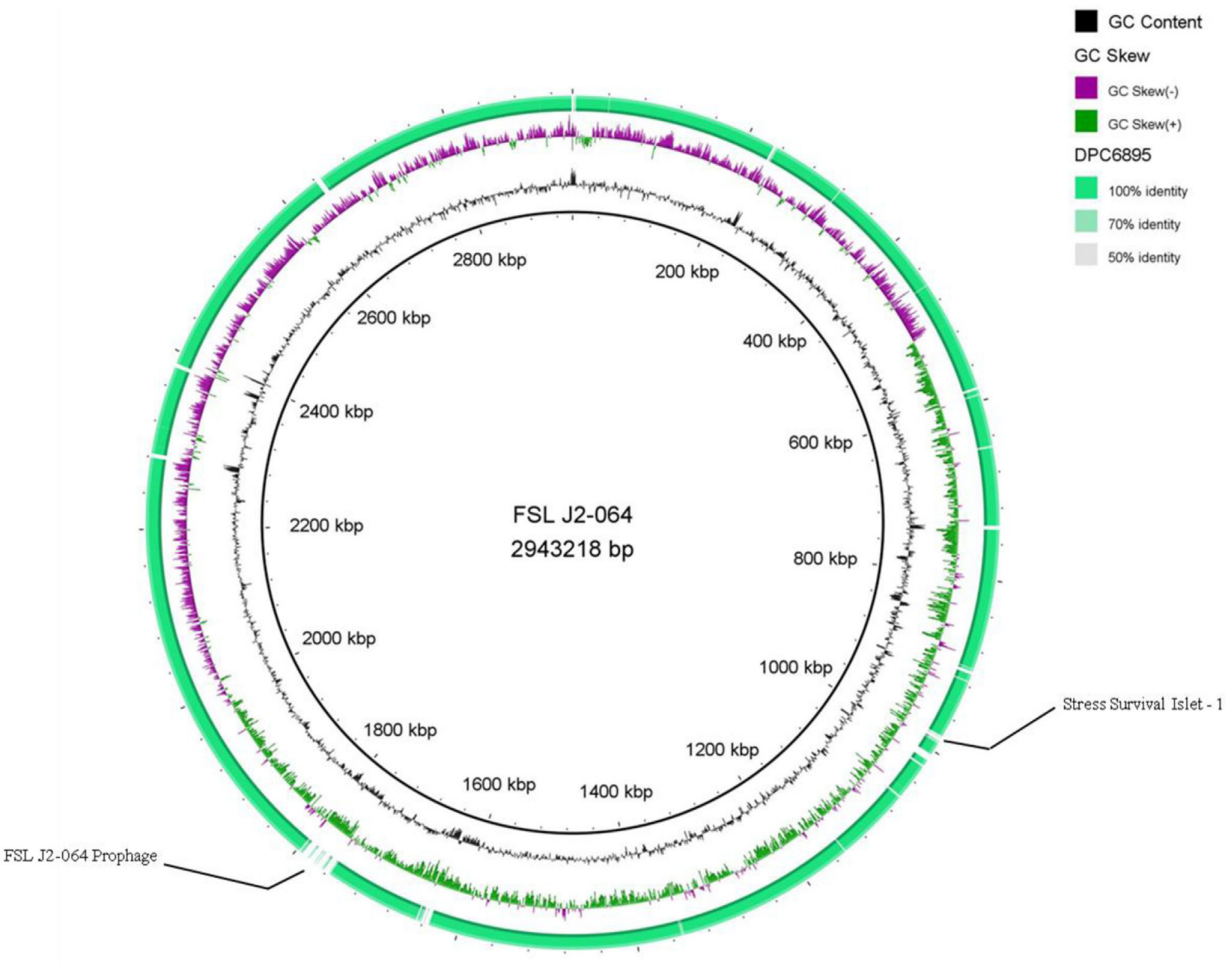
**Circular map of *Listeria monocytogenes* strain DPC6895, using strain FSL J2-064 as a reference genome**. The inner ring denotes the FSL J2-064 genome with corresponding genetic coordinates. The outer ring denotes the genome of strain DPC6895 (green).

Strains DPC6895 and FSL J2-064 were both obtained from bovine sources. While strain FSL J2-064 was originally isolated from a diseased animal (22, 23), strain DPC6895 was notably unable to establish a systemic clinical listeriosis infection in its respective host (20), though this strain did survive numerous antibiotic treatments and continued to be detected in milk expressed from the host for a prolonged period of time. While the host’s immune system may have been a contributing factor, comparative analysis of these two strains was undertaken in order to determine the extent of genetic diversity between them and to identify genomic characteristics, which may account for the observed physiological differences. *L. monocytogenes* strain DPC6895 was determined to contain a total of 123 genes that were not present in the genome of FSL J2-064 (Figure 1; Table S2 in Supplementary Material), while strain FSL J2-064 contained a total of 121 genes that were absent from the DPC6895 genome (Figure 2; Table S3 in Supplementary Material).

### Strain-Specific Genes in *L. monocytogenes* Strain DPC6895

The strain-specific genes in DPC6895 predominantly had functions, which contributed to an enhanced survival ability of this strain in a number of unfavorable environmental conditions. First, a number of these strain-specific genes had annotated functions in bacteriophage resistance. *L. monocytogenes* utilizes a number of biological systems in order to achieve resistance to bacteriophage infection. Foremost among these are the CRISPR sequences together with adjacent Cas genes and the restriction modification (RM) systems, which are widely distributed among prokaryotes (33). CRISPR/Cas genes comprise the adaptive immune system in many bacterial species including *L. monocytogenes* and have a role in defense of the bacterial cell against invading bacteriophages or plasmid-derived elements (34). Immunity against foreign invasion in bacteria is achieved first by integration of a small piece of viral or plasmid DNA (known as a spacer sequence) into the CRISPR locus. During infection, CRISPR-RNAs are transcribed, which guide the Cas proteins to target DNA that matches these spacer sequences that are then cleaved (35). RM systems are also used by bacteria in order to protect themselves from foreign invading DNA, of which there are three distinct classical types in addition to several atypical systems, which differ from one another in terms of their composition and cofactor requirements (36). Each of the different classical RM system types have been previously observed in *L. monocytogenes* (12, 37–39). Strain DPC6895 contained one CRISPR cluster, which consisted of 22 highly conserved direct repeat (DR) regions interspersed with 21 spacer sequences (Table 2). Flanking this CRISPR cluster were a total of seven open reading frames (ORFs) with annotated functions such as Cas genes. BLASTn analyses of the spacer sequences identified homologies to a number of different temperate serovar 1/2-specific *L. monocytogenes* phages including A006, A118, and LP-101, suggesting that this system has a functional role in resistance to infection from these particular siphoviruses. These homologies indicate a role for this particular system in expanding the immunity of these two particular strains to cover a range of both lytic and temperate *L. monocytogenes* phages. The presence of this CRISPR/Cas system may enhance the capacity of strain DPC6895 to withstand a wider array of extracellular threats posed by bacteriophages. No definitive CRISPR/Cas systems were detected in strain FSL J2-064; however, this strain contained all three subunits of a type I RM system, suggesting a difference between these strains in terms of their mechanisms of phage resistance. Strain DPC6895 contains two genes with 100% nucleotide identity to the R and M subunits of this system in FSL J2-064 but does not harbor the third S subunit, and as such this system is presumed to be non-functional in strain DPC6895.

**Table 2:** **Clustered regularly interspaced short palindromic repeat (CRISPR)/CRISPR-associated (Cas) systems in each of the *Listeria monocytogenes* isolates**.

Strain	No. of CRISPR clusters	Location(s) on genome	(F/R)	Conserved region [direct repeat (DR)] consensus sequence	DR length	No. of spacer sequences	No. of flanking Cas genes
DPC6895	1	49,584–50,975 (contig 3)	F	GTTTTAACTACTTATTATGAAATGTAAAT	29	21	7
FSL J2-064	–	–	–	–	–	–	–

Second, a number of the strain-specific genes in DPC6895 had annotated functions associated with antibiotic and heavy metal resistance. *L. monocytogenes* has previously been demonstrated to have quite a broad spectrum of resistance to numerous antibiotics and antimicrobial agents (2, 40) in addition to exhibiting an elevated tolerance to heavy metals (41). The two strains in this study were analyzed for the presence of antibiotic and antimicrobial resistance genes and for heavy metal transporters. The results of this analysis (Table S4 in Supplementary Material) demonstrated that each of the genomes harbor specific resistance genes to a number of antibiotics, including the β-lactams, quinolone, fosfomycin, lincomycin, vancomycin, and tetracycline. Additionally, these strains also contained a number of antimicrobial and quaternary ammonium compound resistance genes including *mdrL* and *lde*, which are believed to be associated with increased tolerance of *L. monocytogenes* to benzalkonium chloride (42). Furthermore, *lde* is also thought to function in resistance of *L. monocytogenes* to fluoroquinolone (43). While both strains encoded many non-specific multidrug resistance transporters, strain DPC6895 harbored one additional multidrug transporter (locus tag *TZ05_2661c*), which was absent from strain FSL J2-064. The exact function of this particular transporter has not yet been fully elucidated, but subsequent BLASTp analysis identified a conserved domain within the translated protein product of this gene, which has a putative function in resistance to the lantibiotic gallidermin (44), suggesting a potentially similar role for this gene in each of these strains. As previously stated, *L. monocytogenes* strain DPC6895 was originally isolated from raw milk expressed by a cow with subclinical bovine mastitis. Following the confirmation of a subclinical infection, the infected cow was medically treated with subsequent intramammary injections of Synulox LC, Tylosin, and oxytetracycline. However, the infected animal’s milk continued to test positive for *L. monocytogenes* despite the intervention with antibiotic treatments (20). Synulox LC contains the antibiotics clavulanic acid and amoxicillin, which are both β-lactams, while oxytetracycline is an antibiotic that is related to tetracycline, and tylosin is a macrolide antibiotic. Analysis of the DPC6895 genome identified a total of eight genes encoding proteins with associated functions in resistance to β-lactams, while a gene associated with tetracycline resistance was also identified. The presence of the aforementioned *mdrL* gene in strain DPC6895 may account for its perceived resistance to Tylosin, given that previous research has demonstrated that disruption of this particular gene resulted in a higher susceptibility to macrolides (45).

**Figure 3:**
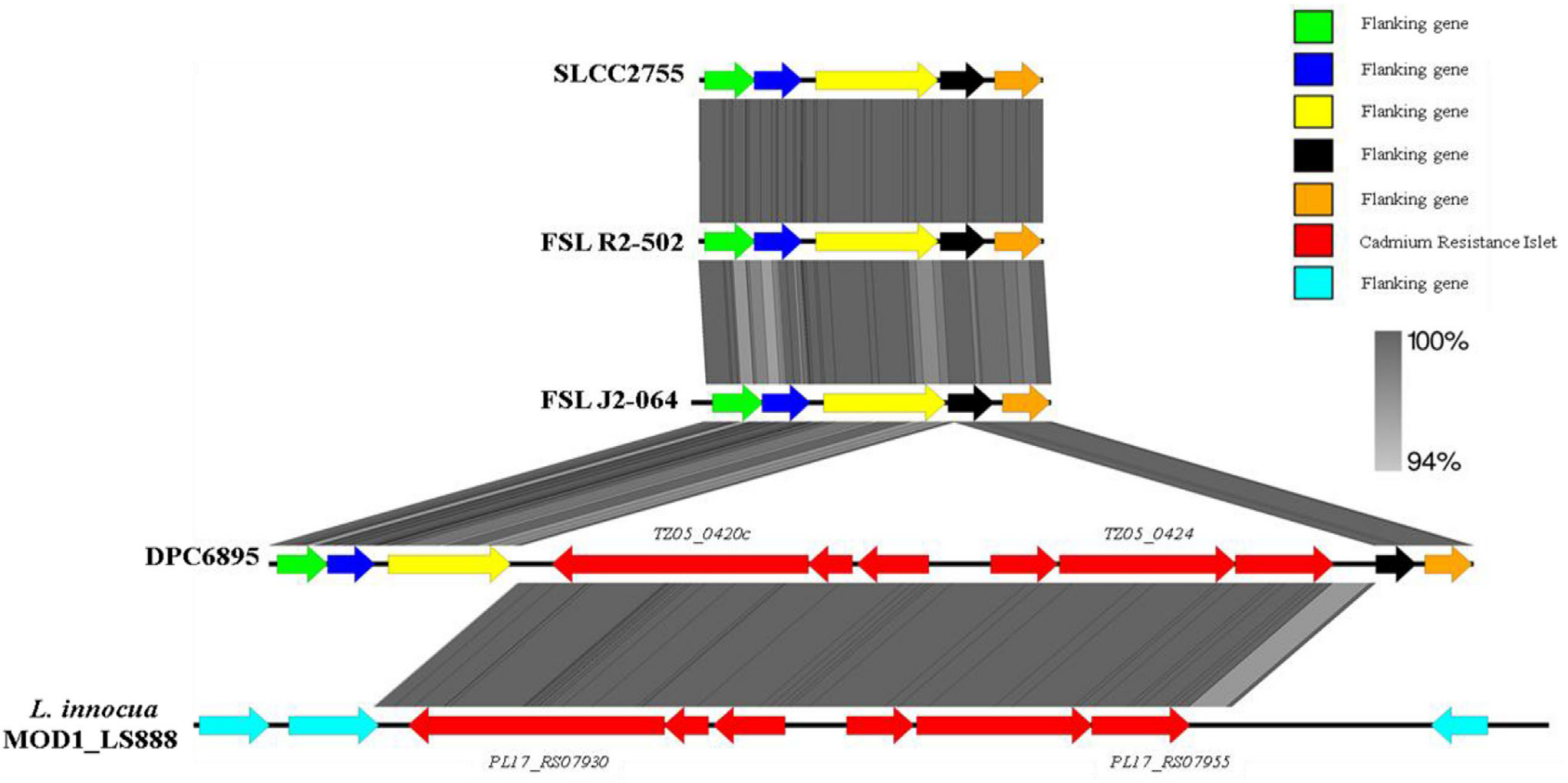
**A linear comparison of the cadmium resistance islet identified in strain DPC6895 with the corresponding genomic region from strain FSL J2-064, two other serotype 1/2b *Listeria monocytogenes* isolates, and an additional *Listeria innocua* isolate, indicating the % amino acid identities between proteins in these clusters**.

In terms of heavy metal resistance, both of the strains contained a number of non-specific heavy metal transporters and specific lead/cadmium/zinc resistance genes. Interestingly, strain DPC6895 was also found to contain a novel 6.5 kb “islet” consisting of six genes, which included a heavy metal transporting ATPase, and a cadmium efflux system accessory protein (Table S5 in Supplementary Material). The G + C content of this islet was 35.9%, indicating that it is possibly of plasmid origin. This six-gene islet has not previously been observed in *L. monocytogenes*, with the only known *Listeria* homologs to these genes found in the recently sequenced *L. innocua* strain MOD1_LS888 (46). Linear alignments between these genomic regions identified a shared 99% nucleotide sequence identity, while also demonstrating that this islet is absent in strain FSL J2-064 and two other *L. monocytogenes* serotype 1/2b genomes that were available on Genbank (Figure 3). The product of one of the genes in this cluster, namely *TZ05_0424*, shares 100% amino acid sequence identity with the *Staphylococcus aureus* transposase Tn552 (47), suggesting strain DPC6895 may have acquired this cadmium resistance islet through a horizontal gene transfer event.

Finally, a number of the strain-specific genes in DPC6895 had annotated functions associated with peptide transport. The oligopeptide permease (*opp*) operon in *L. monocytogenes* consists of five genes (*oppA, oppB, oppC, oppD*, and *oppF*) that are essential for growth at low temperatures and contribute to intracellular growth of this bacterium (48). Comparative analysis of each of the isolates in this study identified the presence of this operon in both of the genomes (data not shown). However, in addition to the oligopeptide transporter operon, strain DPC6895 also contained a unique 5 kb cluster of genes (*TZ05_2018–2022*) within hypervariable hotspot 9, which BLASTp analysis indicated as a dipeptide transport system (*dppABCDF*) (Table S2 in Supplementary Material). The role of this system in strain DPC6895 is unclear. However, previous research has indicated that the presence of dipeptide transporters may confer a selective advantage on *L. monocytogenes*, given the fact that unlike numerous competing bacteria within an environment, it would not need to expend vast amounts of energy on protease synthesis (49). In addition, the presence of a dipeptide transporter would allow the organism to thrive in food samples, which may be deficient in free amino acids but rich in peptides. The presence of this system in strain DPC6895, therefore, could allow it to proliferate in what would be otherwise considered unfavorable environmental conditions.

### Strain-Specific Genes in *L. monocytogenes* Strain FSL J2-064

The strain-specific genes in FSL J2-064 predominantly had functions, which contribute to enhancing the pathogenicity of this isolate. First, a number of the strain-specific genes in FSL J2-064 had annotated functions associated with virulence. *L. monocytogenes* requires a wide array of genes in order to successfully establish a systemic infection within a host organism. These genes, termed “virulence factors,” have functions in a number of different biological processes throughout the infection cycle, including host interaction, internalization, host defense evasion, and *in vitro* proliferation. A large family of leucine-rich proteins, known as the internalins, are important virulence factors involved in host interaction and internalization of pathogenic strains (50). The internalins are classified into four general types on the basis of their specific surface binding domains (51). Type I are known as the LPXTG internalins due to the presence of this sorting signal motif and are covalently anchored to the bacterial cell surface by another virulence factor known as Sortase A. Type II are the GW and WxL internalins, of which just two members (including *inlB*) have been classified to date. Both members of this subfamily display a C-terminal domain that directs a non-covalent association with the *L. monocytogenes* cell surface (51). Type III internalins lack a cell wall-anchoring domain and are secreted by the bacterium. They are thought to promote the cell-to-cell spread of *L. monocytogenes* by relieving the cortical tension of the host cell and enhancing the ability of the bacterium to protrude into the plasma membrane (52). A fourth type of internalin, which contains an atypical leucine-rich repeat region, has also been recently described (53) with *lmo0460* as the sole representative member. The genomes of both strains in this study were examined for the presence of internalin and internalin-like genes. A similar complement of internalins was observed in each input strain (Table S6 in Supplementary Material). Strain FSL J2-064 contained 20 type I internalins, while strain DPC6895 contains 21 (*TZ05_2026c* is novel to this strain). In addition, both isolates contained a virtually identical set of type II and type III internalins. Five homologs of the *L. monocytogenes* strain EGDe type IV internalin *lmo0460* were identified in strain FSL J2-064, localized within hypervariable hotspots 7 and 9. Though the precise function of these type IV internalin proteins has yet to be fully established, they are known to be present in many strains of *L. monocytogenes*, but absent from non-pathogenic *Listeria* species such as *L. innocua*, and thus may have a role in *L. monocytogenes* virulence. Interestingly, strain DPC6895 lacked any identifiable homolog to the recently described type IV internalin of *L. monocytogenes*, and as such, the absence of a type IV internalin in strain DPC6895 may be a contributing factor to its perceived attenuated virulence. Further research, however, is necessary to fully establish their functional role in infection. Additionally, two homologs of the internalin-like gene *lmo0463* of *L. monocytogenes* strain EGDe (hypervariable hotspots 7 and 9, respectively) were identified in strain FSL J2-064 but were absent once again from strain DPC6895. Likewise, their precise role in *L. monocytogenes* virulence remains unclear.

**Figure 4:**
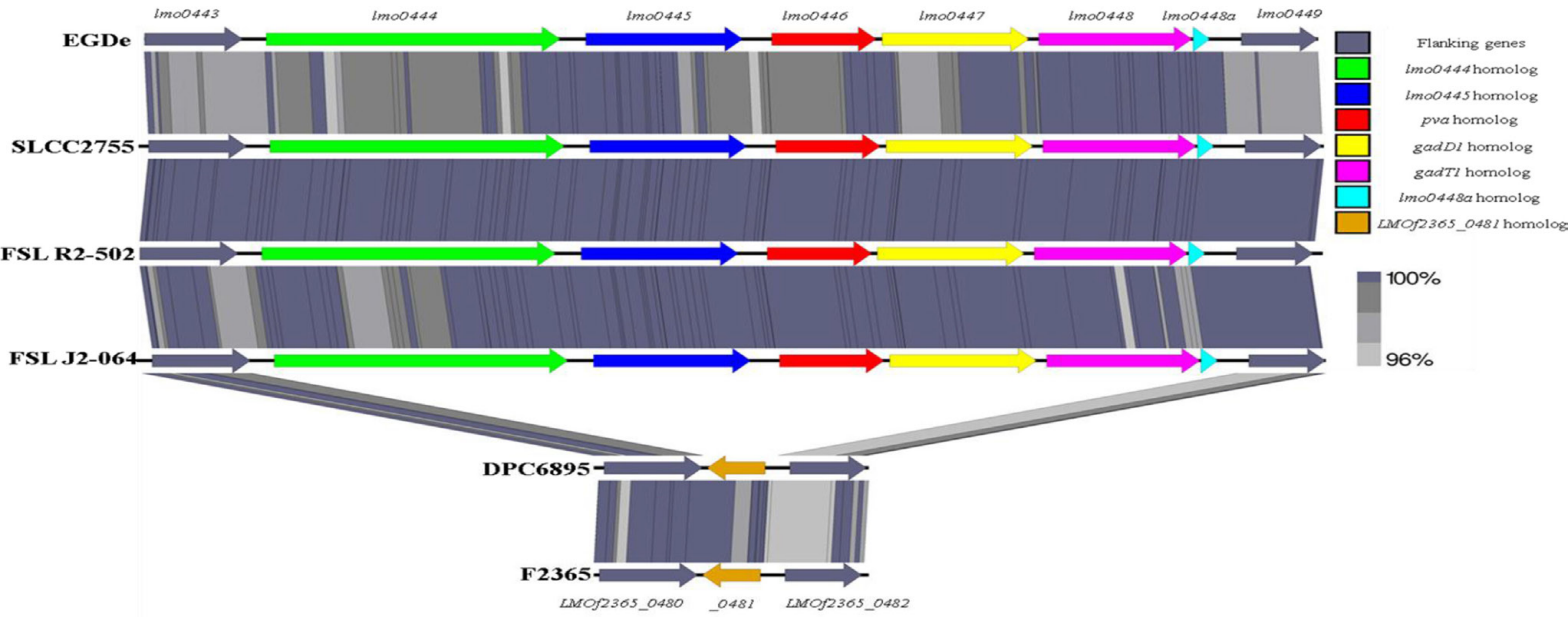
**Linear comparison of the stress survival islet-1 in six different *Listeria monocytogenes* strains, with the % amino acid identities between proteins in these clusters indicated**. The islet is present in strain FSL J2-064, along with the other serotype 1/2b strains FSL R2-502 and SLCC2755, but is absent from strain DPC6895. Instead, the corresponding genomic region in strain DPC6895 displays homology to gene *LMOf2365_0481* of *L. monocytogenes* strain f2365.

Second, the stress survival islet (SSI-1) of *L. monocytogenes* was identified to be present in the genome of strain FSL J2-064 but is absent from that of strain DPC6895 (Figure 4). This islet is an 8.7 kb region of DNA located between *lmo0443* and *lmo0449* of strain EGDe and contains five genes that have been previously implicated to assist in the survival of the bacterium under suboptimal environmental conditions (54). Included within this cluster are the *pva* gene, which has a role in resistance of *L. monocytogenes* to acute toxicity from bile and bile salts (55), and the *gadD1* and *gadT1* genes, which contribute to the efficient growth of the bacterium at low pH (56). The corresponding region in strain DPC6895 was identified to contain a single gene encoding a protein that is 100% identical to LMOf2365_0481 of *L. monocytogenes* serotype 4b strain F2365. The presence of this 182 amino acid protein in strain DPC6895 represents a common feature of islet-negative strains of *L. monocytogenes* (54). Although this particular gene has been observed to be highly conserved within islet-negative strains of *L. monocytogenes*, the function of its hypothetical protein product has not yet been established. Prior research has shown that strains, which do not harbor SSI-1 were observed to grow less efficiently under acidic stresses (12), suggesting that the presence of this particular islet within the genome is beneficial in promoting survival in unfavorable acidic environments posed by the stomach and intestinal tract of a host organism. Additionally, the observed differences in growth efficiency between islet-positive and islet-negative strains of *L. monocytogenes* indicate that the presence of this islet could confer an enhanced ability to proliferate within an organism and lead to an overall higher rate of infection. Therefore, the absence of SSI-1 in DPC6895 may have been a contributing factor in the observed inability of this isolate to establish a clinical infection in its host (20). A number of the strain-specific genes in FSL J2-064 also had annotated functions associated with iron uptake that were absent in strain DPC6895, including the twin-arginine translocase system (57). Given the known association between iron uptake and *L. monocytogenes* virulence (58), the absence of these genes in strain DPC6895 provides another potential insight into its inability to cause a systemic infection.

Finally, the genome of strain FSL J2-064 contained an intact copy of the *comK* gene, while a prophage insertion (contig 7, position 257959–311605) interrupted the *comK* gene in strain DPC6895. The entire *comK* gene sequence in DPC6895 is instead represented by two separate ORFs; *TZ05_2272* and *TZ05_2336*, which together share 100% nucleotide sequence identity with the N- and C-terminal regions of the *comK* gene in FSL J2-064, respectively. A prophage insertion into the *comK* gene of *L. monocytogenes* is a common observation, as this gene represents a “hotspot” for integration of the serotype 1/2-specific bacteriophage A118 and other related phages (11, 13, 59). The phage insertion into this gene may hold downstream consequences for the pathogenic potential of strain DPC6895, as the *comK* gene has recently been shown to have an important role in phagosomal escape of *L. monocytogenes* during infection (60). As such, this interruption to the *comK* gene may be a contributing factor to the attenuated virulence of strain DPC6895. Interestingly however, the same research demonstrated that the *comK* prophage in *L. monocytogenes* strain 10403S excises during bacterial phagocytosis resulting in a reactivation of this gene and the production of a functional ComK protein product, and such an occurrence, therefore, must also be considered a possibility in strain DPC6895. Further investigation is required in order to fully understand the consequences of this prophage insertion.

### Influence of Hypervariable Hotspots on the Virulence of Strains DPC6895 and FSL J2-064

**Figure 5:**
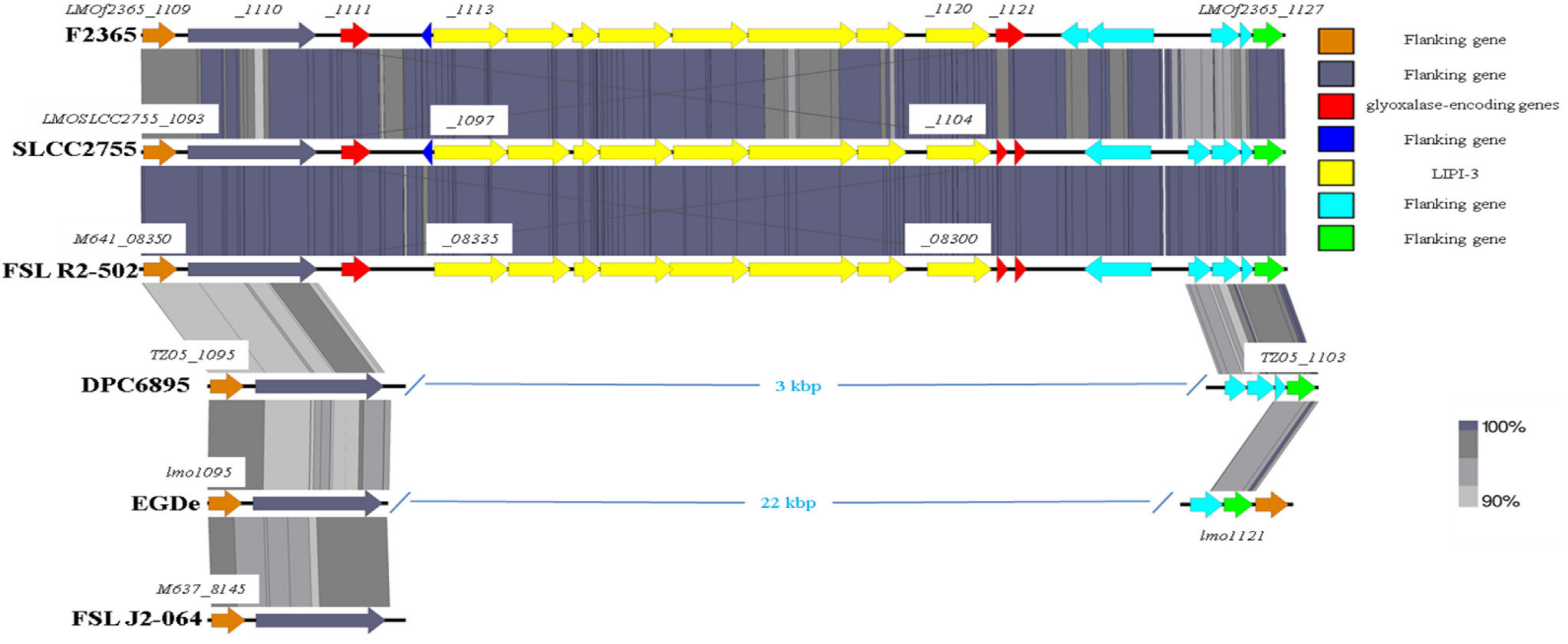
**Comparison of the *Listeria* pathogenicity island 3 between the two bovine isolates and a number of other *L. monocytogenes* strains**.

As previously mentioned, the absence of type IV internalins (all of which are located within hypervariable hotspots in the *L. monocytogenes* genome) may be a contributory factor to the inability of strain DPC6895 to establish a systemic infection in the host. In addition, *Listeria* pathogenicity island 3 (LIPI-3) is a relatively recently discovered pathogenicity island, which has been identified in a subset of atypical *L. innocua* isolates (61) and a number of lineage I strains of *L. monocytogenes*. LIPI-3 contributes to virulence and intracellular survival of the pathogen (62) and is located within hypervariable hotspot 8. The main function of this island is the production of a second *L. monocytogenes* hemolysin, namely listeriolysin S, which is induced under oxidative stress conditions (62). LIPI-3 consists of eight *lls* genes flanked on either side of the island by two related glyoxalase-encoding genes. Comparative analyses with other serotype 1/2b strains of *L. monocytogenes* identified that LIPI-3 was not found in either of the bovine isolates DPC6895 or FSL J2-064 (Figure 5), though homologs of the flanking glyoxalase-encoding genes were identified. The high variability generally observed within hypervariable hotspots of the *L. monocytogenes* genome may account for the absence of this island in these strains. While the presence of LIPI-3 does not appear to be essential in order to establish a systemic infection, the absence of this island may hinder the ability of a particular strain in the establishment of a systemic infection within the host.

## Conclusion

The results of this study demonstrate the high degree of variability that exists between the accessory genomes of closely related *L. monocytogenes* isolates. The hypervariable hotspots found in various areas of the genome may be crucial in defining the physiological characteristics of a particular strain, as evidenced by the presence of important gene clusters such as the type IV internalins and the LIPI-3 within these regions. *L. monocytogenes* strain DPC6895 was shown to be missing some of the key factors that are associated with *in vivo* survival and virulence, including SSI-1 and LIPI-3, providing insights into the inability of this strain to establish a systemic infection in its host. The results highlight a number of potentially crucial factors for *L. monocytogenes* virulence within the accessory genome and suggest that bacterial pathogenesis in *L. monocytogenes* relies on the cumulative effect of a number of genetic factors rather than any single attribute alone. From a regulatory perspective, differentiation of virulent from non-virulent strains is crucially important. As used in this study, whole genome sequencing can be employed as a tool to explore this differentiation.

## Author Contributions

ACasey carried out the laboratory work; KJ, OM, ACoffey, ACasey, and EF were involved in obtaining funding, designing the experiments, interpreting the results, and writing the manuscript.

## Conflict of Interest Statement

The authors declare that the research was conducted in the absence of any commercial or financial relationships that could be construed as a potential conflict of interest.
