# Supplementary material for: Comparative Genomic Analysis of Two Serotype 1/2b Listeria monocytogenes Isolates from Analogous Environmental Niches Demonstrates the Influence of Hypervariable Hotspots in Defining Pathogenesis
Source: Front Nutr. 2016 Dec 21;3:54. doi: 10.3389/fnut.2016.00054 (PMC5174086; doi:10.3389/fnut.2016.00054)
Supplement: Supplementary file 1 [file table_1.pdf]

Table S1: Locations of hypervariable hotspots within each of the input strains.

Hotspot	DPC6895 locus tag range	FSL J2-064 locus tag range
1	TZ05_0070 - TZ05_0084 **(13)**	M637_02925 - M637_02975 **(9)**
2	TZ05_0146 - TZ05_0153c **(6)**	M637_03290 - M637_03345 **(10)**
3	TZ05_0302 - TZ05_0305 **(2)**	M637_04100 - M637_04115 **(2)**
4	TZ05_0310c - TZ05_0323 **(12)**	M637_04140 - M637_04205 **(12)**
5	TZ05_0384 - TZ05_0388c **(3)**	M637_04530 - M637_04560 **(5)**
6	TZ05_0449 - TZ05_0452c **(2)**	M637_04845 - M637_04865 **(3)**
7	TZ05_0471 - TZ05_0478 **(6)**	M637_04985 - M637_05045 **(11)**
8	TZ05_1096 - TZ05_1109 **(12)**	M637_08150 - M637_08240 **(17)**
9	TZ05_2013 - TZ05_2029c **(15)**	M637_13210 - M637_13265 **(10)**
