# Supplementary material for: Comparative Genomic Analysis of Two Serotype 1/2b Listeria monocytogenes Isolates from Analogous Environmental Niches Demonstrates the Influence of Hypervariable Hotspots in Defining Pathogenesis
Source: Front Nutr. 2016 Dec 21;3:54. doi: 10.3389/fnut.2016.00054 (PMC5174086; doi:10.3389/fnut.2016.00054)
Supplement: Supplementary file 2 [file table_2.pdf]

Table S2: Strain-specific genes in *L. monocytogenes* strain DPC6895 when compared to strain FSL J2-064.

Start	End	Contig	F/R	Length (AA)	Locus Tag	Function
77994	78378	1	F	144	*TZ05_0076*	Hypothetical Protein
78796	79512	1	F	238	*TZ05_0077*	Hypothetical Protein
79513	79836	1	F	107	*TZ05_0078*	Hypothetical Protein
80003	80296	1	F	97	*TZ05_0079*	Hypothetical Protein
80433	81155	1	F	240	*TZ05_0080*	Membrane Protein
81395	81577	1	F	60	*TZ05_0081*	Hypothetical Protein
82262	82546	1	F	94	*TZ05_0082*	Hypothetical Protein
82555	82983	1	F	142	*TZ05_0083*	Hypothetical Protein
88623	89021	1	F	132	*TZ05_0089*	Hypothetical Protein
89197	89799	1	F	200	*TZ05_0090*	Hypothetical Protein
165210	165809	2	F	199	*TZ05_0385*	Hypothetical Protein
165806	167164	2	F	452	*TZ05_0386*	Retron-type RNA-directed DNA polymerase
167207	167323	2	F	38	*TZ05_0387*	Hypothetical Protein
198138	196027	2	R	703	*TZ05_0420c*	Lead/cadmium/zinc and mercury transporting ATPase
198499	198131	2	R	122	*TZ05_0421c*	Cadmium efflux system accessory protein / ArsR family transcriptional regulator
199129	198536	2	R	197	*TZ05_0422c*	SAM-dependent methyltransferase
199636	200190	2	F	187	*TZ05_0423*	Resolvase / integrase Bin
200204	201658	2	F	484	*TZ05_0424*	Tn552 transposase
201651	202463	2	F	270	*TZ05_0425*	ATP-binding protein p271
240321	239773	2	R	182	*TZ05_0461c*	Stress Survival Islet gene F2365 homolog
2002	2184	3	F	60	*TZ05_0475*	Hypothetical Protein
2306	3133	3	F	275	*TZ05_0476*	Hypothetical Protein
3161	3520	3	F	119	*TZ05_0477*	Putative Secreted Protein
10368	10745	3	F	125	*TZ05_0486*	Hypothetical Protein
42503	43325	3	F	240	*TZ05_0520*	CRISPR repeat RNA endoribonuclease Cas6

Start	End	Contig	F/R	Length (AA)	Locus Tag	Function
43241	44929	3	F	562	*TZ05_0521*	CRISPR-associated protein Cst1
44919	45788	3	F	289	*TZ05_0522*	CRISPR-associated negative autoregulator
45766	46548	3	F	260	*TZ05_0523*	CRISPR-associated protein Cas5
46616	48805	3	F	729	*TZ05_0524*	CRISPR-associated helicase Cas3
48861	49103	3	F	80	*TZ05_0525*	CRISPR-associated protein Cas1 maturase
49066	49386	3	F	106	*TZ05_0526*	CRISPR-associated protein Cas2
282358	282810	4	F	150	*TZ05_0816*	Transcriptional regulator, MarR family
282826	283332	4	F	168	*TZ05_0817*	GNAT family acetyltransferase
565862	566539	4	F	225	*TZ05_1097*	Hypothetical Protein
566524	567237	4	F	237	*TZ05_1098*	Hypothetical Protein
567350	568159	4	F	269	*TZ05_1099*	Hypothetical Protein
569250	569618	4	F	122	*TZ05_1103*	Hypothetical Protein
1055357	1054485	4	R	290	*TZ05_1587c*	Hypothetical Protein
10191	8575	5	R	538	*TZ05_1682c*	Site-specific recombinase
10425	10249	5	R	58	*TZ05_1683c*	Hypothetical Protein
10891	10631	5	R	86	*TZ05_1684c*	Hypothetical Protein
11434	11171	5	R	87	*TZ05_1685c*	Hypothetical Protein
11539	11754	5	F	71	*TZ05_1686*	Transcriptional Regulator
11958	13352	5	F	464	*TZ05_1687*	RLX protein
14263	13655	5	R	202	*TZ05_1688c*	Hypothetical Protein
15725	14265	5	R	486	*TZ05_1689c*	Hypothetical Protein
39316	38255	5	R	353	*TZ05_1716c*	Hypothetical Protein
3426	4115	7	F	229	*TZ05_2017*	Methyltransferase
4112	5680	7	F	522	*TZ05_2018*	Dipeptide-binding ABC transporter, periplasmic substrate-binding component
5682	6620	7	F	312	*TZ05_2019*	Dipeptide transport system permease protein DppB
6617	7399	7	F	260	*TZ05_2020*	Dipeptide transport system permease protein DppC
7392	8099	7	F	235	*TZ05_2021*	ABC-type nickel / oligopeptides specific transport system, ATPase component
8092	8763	7	F	223	*TZ05_2022*	NAD+ synthetase

Start	End	Contig	F/R	Length (AA)	Locus Tag	Function
9794	9444	7	R	116	*TZ05_2023c*	Hypothetical Protein
10897	10076	7	R	273	*TZ05_2024c*	Hypothetical Protein
11150	11019	7	R	43	*TZ05_2025c*	Hypothetical Protein
12658	11333	7	R	441	*TZ05_2026c*	Internalin-like protein
13007	14509	7	F	500	*TZ05_2027*	Hypothetical Protein
15691	14891	7	R	266	*TZ05_2028c*	Hypothetical Protein
272943	273152	7	F	69	*TZ05_2275*	Hypothetical Protein
273254	273658	7	F	134	*TZ05_2276*	Hypothetical Protein
273660	274088	7	F	142	*TZ05_2277*	Hypothetical Protein
274857	275645	7	F	262	*TZ05_2279*	Phage Protein
276812	276531	7	R	93	*TZ05_2281c*	Phage Holin
277190	276825	7	R	121	*TZ05_2282c*	Hypothetical Protein
280972	279404	7	R	522	*TZ05_2284c*	Phage-related protein
285768	280969	7	R	1599	*TZ05_2285c*	Phage Tail Tape Measure Protein
285949	285773	7	R	58	*TZ05_2286c*	Hypothetical Protein
286512	286081	7	R	143	*TZ05_2287c*	Hypothetical Protein
287254	286568	7	R	228	*TZ05_2288c*	Hypothetical Protein
287630	287259	7	R	123	*TZ05_2289c*	Hypothetical Protein
287944	287627	7	R	105	*TZ05_2290c*	Hypothetical Protein
288299	287934	7	R	121	*TZ05_2291c*	Hypothetical Protein
288652	288299	7	R	117	*TZ05_2292c*	Hypothetical Protein
288820	288653	7	R	55	*TZ05_2293c*	Phage Protein
289706	288834	7	R	290	*TZ05_2294c*	Hypothetical Protein
290283	289729	7	R	184	*TZ05_2295c*	Phage Capsid / Scaffold Protein
291422	290379	7	R	347	*TZ05_2296c*	Hypothetical Protein
292983	291427	7	R	518	*TZ05_2297c*	Hypothetical Protein
294158	292998	7	R	386	*TZ05_2298c*	Phage Terminase
295041	294256	7	R	261	*TZ05_2299c*	Phage Terminase Small subunit

Start	End	Contig	F/R	Length (AA)	Locus Tag	Function
295308	295081	7	R	75	*TZ05_2300c*	Hypothetical Protein
296021	295389	7	R	210	*TZ05_2301c*	Phage Protein
296008	296157	7	R	49	*TZ05_2302c*	Phage Protein
296637	296203	7	R	144	*TZ05_2303c*	Hypothetical Protein
296789	296664	7	R	41	*TZ05_2304c*	Phage Protein
297186	296782	7	R	134	*TZ05_2305c*	endodeoxyribonuclease
297292	297152	7	R	46	*TZ05_2306c*	Hypothetical Protein
297594	297289	7	R	101	*TZ05_2307c*	Phage Protein
298506	298105	7	R	133	*TZ05_2309c*	Hypothetical Protein
298862	298503	7	R	119	*TZ05_2310c*	Hypothetical Protein
299114	298884	7	R	76	*TZ05_2311c*	RNA-binding protein
299238	299080	7	R	52	*TZ05_2312c*	Hypothetical Protein
299780	299250	7	R	176	*TZ05_2313c*	Sugar-phosphate nucleotidyltransferase
300064	299777	7	R	95	*TZ05_2314c*	Hypothetical Protein
301044	300061	7	R	327	*TZ05_2315c*	Hypothetical Protein
301720	301061	7	R	219	*TZ05_2316c*	Putative recombination protein / Single-stranded DNA-binding protein
302393	302199	7	R	64	*TZ05_2318c*	Hypothetical Protein
302617	302489	7	R	42	*TZ05_2319c*	Phage Protein
302887	302699	7	R	62	*TZ05_2320c*	Hypothetical Protein
303211	302996	7	R	71	*TZ05_2321c*	Phage Protein
303741	303208	7	R	177	*TZ05_2322c*	Phage Protein
304641	303865	7	R	258	*TZ05_2323c*	Phage antirepressor protein / Antirepressor
304705	304902	7	F	65	*TZ05_2324*	Adenylate kinase
305185	304904	7	R	93	*TZ05_2325c*	Hypothetical Protein
305418	305182	7	R	78	*TZ05_2326c*	Hypothetical Protein
305483	305842	7	F	119	*TZ05_2327*	Hypothetical Protein
305995	305801	7	R	64	*TZ05_2328c*	Hypothetical Protein
306250	305999	7	R	83	*TZ05_2329c*	DNA-binding protein

Start	End	Contig	F/R	Length (AA)	Locus Tag	Function
306413	306718	7	F	101	*TZ05_2330*	XRE family transcriptional regulator
306749	307240	7	F	163	*TZ05_2331*	Hypothetical Protein
307267	307974	7	F	235	*TZ05_2332*	Hypothetical Protein
308033	308467	7	F	144	*TZ05_2333*	Hypothetical Protein
308672	310186	7	F	504	*TZ05_2334*	ATP-dependent DNA helicase RecG
310247	311605	7	F	452	*TZ05_2335*	Integrase
39996	38089	8	R	635	*TZ05_2412c*	Hypothetical Protein
41923	40007	8	R	638	*TZ05_2413c*	Hypothetical Protein
47631	47149	9	R	160	*TZ05_2660c*	Hypothetical Protein
48272	47628	9	R	214	*TZ05_2661c*	ABC transporter, ATP-binding protein
49033	48284	9	R	249	*TZ05_2662c*	Membrane Protein
49733	49035	9	R	232	*TZ05_2663c*	Membrane Protein
50505	49720	9	R	261	*TZ05_2664c*	Conserved membrane-spanning protein
50978	50571	9	R	135	*TZ05_2665c*	Hypothetical Protein
