# Supplementary material for: Comparative Genomic Analysis of Two Serotype 1/2b Listeria monocytogenes Isolates from Analogous Environmental Niches Demonstrates the Influence of Hypervariable Hotspots in Defining Pathogenesis
Source: Front Nutr. 2016 Dec 21;3:54. doi: 10.3389/fnut.2016.00054 (PMC5174086; doi:10.3389/fnut.2016.00054)
Supplement: Supplementary file 3 [file table_3.pdf]

Table S3: Strain-specific genes in *L. monocytogenes* strain FSL J2-064 when compared to strain DPC6895.

Start	End	Contig	F/R	Length (AA)	Locus Tag	Function
40924	40610	1	R	104	*M637_00225*	Hypothetical Protein
317694	317584	1	R	36	*M637_01675*	Hypothetical Protein
318242	317709	1	R	177	*M637_10680*	MFS transporter
459633	458926	1	R	235	*M637_02355*	Hypothetical Protein
584780	585394	1	F	204	*M637_02955*	Hypothetical Protein
585704	586057	1	F	117	*M637_02960*	Hypothetical Protein
586633	586740	1	F	35	*M637_02970*	Hypothetical Protein
592353	592715	1	F	120	*M637_03005*	Hypothetical Protein
593188	593532	1	F	114	*M637_03010*	Hypothetical Protein
650746	650988	1	F	80	*M637_03310*	Hypothetical Protein
651009	651452	1	F	147	*M637_03315*	Hypothetical Protein
651684	651920	1	F	78	*M637_03320*	Hypothetical Protein (ADP-ribosyl cyclase / cyclic ADP-ribose hydrolase / CD38 domain)
651926	652159	1	F	77	*M637_03325*	Hypothetical Protein (Immunity protein 45 domain)
652247	652594	1	F	115	*M637_03330*	Hypothetical Protein (dihydroorotate dehydrogenase (quinone))
900391	899657	1	R	244	*M637_04450*	Preprotein translocase subunit TatC
900567	900388	1	R	59	*M637_04455*	Preprotein translocase subunit TatA
902444	903928	1	F	494	*M637_04470*	FTR1 family iron permease
903925	905085	1	F	386	*M637_04475*	Hypothetical Protein (Iron transport domain)
905103	906368	1	F	421	*M637_04480*	Deferrochelatase
913938	914078	1	F	46	*M637_04535*	Hypothetical Protein
914117	914563	1	F	148	*M637_04540*	Hypothetical Protein
914655	914906	1	F	83	*M637_04545*	Hypothetical Protein
915000	915284	1	F	94	*M637_04550*	Hypothetical Protein
915521	915907	1	F	128	*M637_04555*	Hypothetical Protein
981302	984082	1	F	926	*M637_04910*	Membrane / Phage Infection Protein

Start	End	Contig	F/R	Length (AA)	Locus Tag	Function
984326	985813	1	F	495	*M637_04915*	Transcriptional Regulator
986087	987076	1	F	329	*M637_04920*	Hypothetical Protein
990079	990234	1	F	51	*M637_04935*	Hypothetical Protein
1003561	1005099	1	F	512	*M637_04990*	Transcriptional Regulator
1005363	1007444	1	F	693	*M637_04995*	Type IV internalin
1007580	1009649	1	F	689	*M637_05000*	Type IV internalin
1009785	1010012	1	F	75	*M637_05005*	Hypothetical Protein
1010109	1011542	1	F	477	*M637_05010*	Type IV internalin
1011608	1012081	1	F	157	*M637_05015*	Hypothetical Protein
1012101	1012586	1	F	161	*M637_05020*	Hypothetical Protein
1012614	1012919	1	F	101	*M637_05025*	*lmo0463* homolog
1019364	1021127	1	F	587	*M637_05070*	Reverse Transcriptase
1060772	1061959	1	F	395	*M637_05265*	Hypothetical Protein
1062044	1062973	1	F	309	*M637_05270*	Integrase
1064230	1063013	1	R	405	*M637_05275*	Type I restriction endonuclease subunit S
1147280	1145916	1	R	454	*M637_05655*	Cell Wall Anchor Protein
1349581	1350012	1	F	143	*M637_06730*	Hypothetical Protein
1350090	1350362	1	F	90	*M637_06735*	Hypothetical Protein
1633335	1635710	1	F	791	*M637_08155*	ATP-dependent DNA helicase PcrA
1635733	1637316	1	F	527	*M637_08160*	Hypothetical Protein
1637301	1638419	1	F	372	*M637_08165*	Hypothetical Protein (UvrD domain)
1638443	1639534	1	F	363	*M637_08170*	Hypothetical Protein
1640028	1640843	1	F	271	*M637_08175*	Nucleotidyltransferase
1640864	1641421	1	F	185	*M637_08180*	Hypothetical Protein
1641849	1641568	1	R	93	*M637_08185*	Transcriptional Regulator
1642748	1644145	1	F	465	*M637_08200*	Hypothetical Protein (LXG domain containing)
1646345	1646848	1	F	167	*M637_08225*	Hypothetical Protein
1646962	1647264	1	F	100	*M637_08230*	Hypothetical Protein

Start	End	Contig	F/R	Length (AA)	Locus Tag	Function
1647407	1647937	1	F	176	*M637_08235*	Hypothetical Protein (Protein WWC2)
1755467	1754313	1	R	384	*M637_08830*	Integrase
1756174	1755602	1	R	190	*M637_08835*	Hypothetical Protein
1756677	1756225	1	R	150	*M637_08840*	Toxin
1757017	1756694	1	R	107	*M637_08845*	XRE family transcriptional regulator
1757301	1757477	1	F	58	*M637_08850*	Hypothetical Protein
1757464	1757616	1	F	50	*M637_08855*	Hypothetical Protein
1757928	1757635	1	R	97	*M637_08860*	Hypothetical Protein
1757998	1758321	1	F	107	*M637_08865*	Hypothetical Protein
1758336	1758539	1	F	67	*M637_08870*	XRE family transcriptional regulator
1758541	1758783	1	F	80	*M637_08875*	Hypothetical Protein
1758786	1758971	1	F	61	*M637_08880*	Hypothetical Protein
1759074	1759196	1	F	40	*M637_08885*	Hypothetical Protein
1759206	1759358	1	F	50	*M637_08890*	Hypothetical Protein
1759495	1760208	1	F	237	*M637_08895*	Hypothetical Protein
1760219	1761163	1	F	314	*M637_08900*	Integrase
1761176	1761856	1	F	226	*M637_08905*	Hypothetical Protein (Myb-like DNA-binding domain)
1761853	1762422	1	F	189	*M637_08910*	Phage Related Protein
1762419	1762943	1	F	174	*M637_08915*	Hypothetical Protein
1762940	1763530	1	F	196	*M637_08920*	Hypothetical Protein
1763530	1763733	1	F	67	*M637_08925*	Hypothetical Protein
1763734	1763934	1	F	66	*M637_08930*	Hypothetical Protein
1763931	1764431	1	F	166	*M637_08935*	Hypothetical Protein
1765037	1764477	1	R	186	*M637_08940*	Hypothetical Protein
1765142	1765315	1	F	57	*M637_08945*	Hypothetical Protein
1765312	1765695	1	F	127	*M637_08950*	Hypothetical Protein
1766195	1766887	1	F	230	*M637_08960*	DNA-binding Protein
1766951	1768207	1	F	418	*M637_08965*	Helicase

Start	End	Contig	F/R	Length (AA)	Locus Tag	Function
1768232	1768717	1	F	161	*M637_08970*	Hypothetical Protein
1768740	1771082	1	F	780	*M637_08975*	DNA Primase
1771378	1771698	1	F	106	*M637_08980*	Hypothetical Protein
1771695	1771964	1	F	89	*M637_08985*	Hypothetical Protein
1771967	1772605	1	F	212	*M637_08990*	Hypothetical Protein
1772606	1773031	1	F	141	*M637_08995*	Hypothetical Protein
1773261	1774160	1	F	299	*M637_09000*	Hypothetical Protein
1774421	1774747	1	F	108	*M637_09005*	Hypothetical Protein
1774747	1775061	1	F	104	*M637_09010*	alpha/beta hydrolase
1775167	1775466	1	F	99	*M637_09015*	Hypothetical Protein
1775463	1777106	1	F	547	*M637_09020*	Terminase
1777505	1777116	1	R	129	*M637_09025*	Hypothetical Protein
1777556	1778686	1	F	376	*M637_09030*	Portal Protein
1778683	1779399	1	F	238	*M637_09035*	Peptidase
1779426	1780577	1	F	383	*M637_09040*	Phage Capsid Protein
1780584	1780754	1	F	56	*M637_09045*	Hypothetical Protein
1780764	1781063	1	F	99	*M637_09050*	Hypothetical Protein
1781047	1781412	1	F	121	*M637_09055*	Phage head-tail adapter Protein
1781409	1781810	1	F	133	*M637_09060*	Hypothetical Protein
1781807	1782190	1	F	127	*M637_09065*	Hypothetical Protein
1782211	1782798	1	F	195	*M637_09070*	Phage tail protein
1782869	1783201	1	F	110	*M637_09075*	Hypothetical Protein
1783252	1783413	1	F	53	*M637_09080*	Hypothetical Protein
1783417	1788336	1	F	1639	*M637_09085*	Phage Tail Tape Measure Protein
1788329	1789978	1	F	549	*M637_09090*	Phage Tail Protein
1789991	1792285	1	F	764	*M637_09095*	Hypothetical Protein
1793419	1793862	1	F	147	*M637_09105*	Hypothetical Protein
1793841	1794257	1	F	138	*M637_09110*	Hypothetical Protein

Start	End	Contig	F/R	Length (AA)	Locus Tag	Function
1794278	1794544	1	F	88	*M637_09115*	Phage Holin
1796444	1795995	1	R	149	*M637_09130*	Hypothetical Protein
1796821	1796450	1	R	123	*M637_09135*	Hypothetical Protein
1797083	1796850	1	R	77	*M637_09140*	Hypothetical Protein
1797617	1797778	1	F	53	*M637_09150*	Hypothetical Protein
2641107	2640802	1	R	101	*M637_13220*	*lmo0463* homolog
2641620	2641135	1	R	161	*M637_13225*	Hypothetical Protein
2642113	2641640	1	R	157	*M637_13230*	Hypothetical Protein
2644248	2642179	1	R	689	*M637_13235*	Type IV internalin
2646452	2644383	1	R	689	*M637_13240*	Type IV internalin
2648232	2646694	1	R	512	*M637_13245*	Transcriptional Regulator
2650242	2650397	1	F	51	*M637_13260*	Hypothetical Protein
