# Supplementary material for: Comparative Genomic Analysis of Two Serotype 1/2b Listeria monocytogenes Isolates from Analogous Environmental Niches Demonstrates the Influence of Hypervariable Hotspots in Defining Pathogenesis
Source: Front Nutr. 2016 Dec 21;3:54. doi: 10.3389/fnut.2016.00054 (PMC5174086; doi:10.3389/fnut.2016.00054)
Supplement: Supplementary file 4 [file table_4.pdf]

Table S4: Antibiotic / Heavy Metal resistance genes in each input *L. monocytogenes* strain

Function	DPC6895 locus tag<br>Prefix: TZ05_	FSL J2-064 locus tag<br>Prefix: M637_
**Multi-drug Resistance**	0003, 0116c, 0117c, 0615c, 0617, 0681, 0752, 0836, 0984c, 0993, 1607, 1639, 1640, 2072, **2661c,** 2741, 2744, 2764, 2771, 2797	01905, 01920, 02025, 02060, 02190, 02590, 03140, 03145, 05700, 05710, 06035, 06395, 06830, 07585, 07630, 11065, 11230, 11235, 13485
**Benzalkonium Chloride resistance *mdrL***	1398	10025
**Benzalkonium Chloride resistance *lde***	2760c	2005
**Bacteriocin Transport & Multi Antimicrobial extrusion**	1842, 1898c, 2086c	12340, 12620, 13560
**Tellurite Resistance**	1962	12945
**Camphor Resistance**	2080c, 2081c	13525, 13530
**Bacitracin Transport**	0031c, 1626, 1744c, 2114	02730, 11160, 11845, 13700
**QAC Resistance**	0852, 0853	06910, 06915
**β-lactamase & Metallo-beta-lactamase proteins**	0298, 0946, 1423c, 1567c, 1604c, 1787, 2157c, 2167c	04090, 07400, 10150, 10870, 11050, 12070, 13940, 13990
**Quinolone Resistance**	1240, 2836	02380, 09215
**Fosfomycin Resistance**	1701c	11630
**Vancomycin Resistance**	1643, 1695	11260, 11600
**Aminoglycoside N3-acetyltransferase**	1707	11660
**Lincomycin Resistance**	0528, 2596c	05245, 01145
**Tetracycline Resistance**	0838c	6840
**Heavy Metal Transport**	1848c, 2061, 1435c	13430, 12370, 10210
**Aluminium Resistance**	1287	9465
**Lead / Cadmium / Zinc Resistance**	**0420c, 0421,** 0652, 1849c, 2233, 2426c, 2582c	05890, 12375, 14315, 00290, 01080
