# Supplementary material for: Comparative Genomic Analysis of Two Serotype 1/2b Listeria monocytogenes Isolates from Analogous Environmental Niches Demonstrates the Influence of Hypervariable Hotspots in Defining Pathogenesis
Source: Front Nutr. 2016 Dec 21;3:54. doi: 10.3389/fnut.2016.00054 (PMC5174086; doi:10.3389/fnut.2016.00054)
Supplement: Supplementary file 5 [file table_5.pdf]

Table S5: *L. monocytogenes* strain DPC6895 Cadmium Resistance Islet

Start	End	Contig	Length (Amino Acids)	Locus Tag	Function
198138	196027	2	703	*TZ05_0420c*	Lead/cadmium/zinc and mercury transporting ATPase
198499	198131	2	122	*TZ05_0421c*	Cadmium efflux system accessory protein / ArsR family transcriptional regulator
199129	198536	2	197	*TZ05_0422c*	SAM-dependent methyltransferase
199636	200190	2	187	*TZ05_0423*	Resolvase/Integrase Bin
200204	201658	2	484	*TZ05_0424*	Tn552 transposase
201651	202463	2	270	*TZ05_0425*	ATP-binding protein p271
