# Supplementary material for: Comparative Genomic Analysis of Two Serotype 1/2b Listeria monocytogenes Isolates from Analogous Environmental Niches Demonstrates the Influence of Hypervariable Hotspots in Defining Pathogenesis
Source: Front Nutr. 2016 Dec 21;3:54. doi: 10.3389/fnut.2016.00054 (PMC5174086; doi:10.3389/fnut.2016.00054)
Supplement: Supplementary file 6 [file table_6.pdf]

Table S6: A list of internalin genes present in each of the *L. monocytogenes* input strains.

Internalin Family	Gene Name	EGDe homolog	DPC6895 locus tag	FSL J2-064 locus tag
Type I LPXGT-internalins	*inlA*	*lmo0433*	TZ05_0450	M637_04850
	inlC2/H	*lmo0263*	TZ05_0268	M637_03935
	inlD	-	TZ05_0269	M637_03940
	inlE	*lmo0264*	TZ05_0270	M637_03945
	inlF	*lmo0409*	TZ05_0429	M637_04750
	inlI	*lmo0333*	TZ05_0340	M637_04290
	inlJ	*lmo2821*	TZ05_2839	M637_02395
	inlK	*lmo1290*	TZ05_1280	M637_09430
		lmo0327	TZ05_0336	M637_04270
		lmo0331	TZ05_0338c	M637_04280
		lmo0514	TZ05_0516	M637_05220
		lmo0610	TZ05_0620c	M637_05725
		lmo0732	TZ05_0746	M637_06365
		lmo1136	TZ05_1121	M637_08305
		lmo2026	TZ05_0790	M637_06585
		lmo2396	TZ05_2401	M637_00170
		-	TZ05_0339	M637_04285
		-	TZ05_0364	M637_04410
		-	TZ05_1230	M637_09165
		-	TZ05_2026c	-
		-	TZ05_2672	M637_01530
Type II GW-/WxL-internalins	*inlB*	*lmo0434*	TZ05_0451	M637_04855
		lmo0549	TZ05_0559	M637_05415

Internalin Family	Gene Name	EGDe homolog	DPC6895 locus tag	FSL J2-064 locus tag
Type III Secreted internalins	*inlC*	*lmo1786*	TZ05_1783c	M637_12050
		lmo2445	TZ05_2449c	M637_00405
		lmo2470	TZ05_2474	M637_00545
		lmo0460	-	M637_04995
		lmo0460	-	M637_05000
Type IV internalins		*lmo0460*	-	M637_05010
		lmo0460	-	M637_13235
		lmo0460	-	M637_13240
		lmo0460	-	
Internalin-like proteins		*lmo0463*	-	M637_05025
		lmo0463	-	M637_13220
		-	TZ05_0276c	M637_03975
		-	TZ05_2447c	M637_00395
